# Reconstruction of complicated spinal tuberculosis with long-segment fibula transplantation: a case report

**DOI:** 10.1186/s12891-023-06935-4

**Published:** 2023-10-17

**Authors:** RuiYang Wang, FeiFan Wang, Qing Liu, Fan Zhang, JianFeng Chen, Bin Wu, Neng Ru

**Affiliations:** Orthopedics Department, the First College of Clinical Medical Science, China Three Gorges University, Yichang, China

**Keywords:** Spinal tuberculosis, Free fibula graft, Abscess, Debridement, Orthopedic, Kyphosis

## Abstract

**Background:**

Treating complex cases of spinal tuberculosis (STB) that involve multiple vertebral bodies and cause destruction of the spinal structure, kyphotic deformity, and acute nerve injury can be challenging. This report describes the course of treatment and 5-year follow-up of a complex case of multisegmental STB.

**Case presentation:**

This report describes a case of tuberculosis affecting the vertebrae extending from thoracic 12 to lumbar 5 in a 60-year-old woman who suffered sudden paralysis in both lower extremities. The patient underwent emergency posterior paraspinal abscess clearance, laminectomy with spinal decompression. Partial correction of the kyphotic deformity via long-segment fixation from the T9 vertebral body to the ilium in a one-stage posterior procedure. The patient’s neurological status was diagnosed as grade E on the American Spinal Injury Association (ASIA) scale after the one-stage operation. Following standardized 4-combination anti-tuberculosis drug therapy for three months in postoperative patients, the patient underwent two-stage transabdominal anterior abscess removal, partial debridement of the lesion and bilateral fibula graft support. One year after the two-stage operation, the patient’s visual analog scale (VAS) score of back pain was 1 point, and the patient’s erythrocyte sedimentation rate (ESR) and C-reactive protein (CRP) levels returned to normal. Five years after the second-stage operation, the Oswestry disability index (ODI) of patient quality of life was 14 points. There was a 4-degree change in the Cobb angle over five years. During the five-year follow-up period, the grafted fibula did not experience any subsidence.

**Conclusion:**

For patients with spinal tuberculosis and acute paralysis, it is essential to relieve spinal cord compression as soon as possible to recover spinal cord function. For lesions that cannot be debrided entirely, although limited debridement combined with anti-tuberculosis drug therapy has the risk of sinus formation and tuberculosis recurrence, it is much safer than the risk of thorough debridement surgery. In this case, an unconventional long-segment fibula graft, pelvis-vertebral support, was an effective reconstruction method.

## Introduction

When spinal tuberculosis is combined with complications such as nerve damage, spinal instability, and kyphosis, surgical intervention is often needed. The purpose is to restore the sequence and stability of the spine and restore spinal cord function to the greatest extent [1, 2]. “Complete” clearance of TB lesions reduces recurrence rates and prevents sinus tract formation [3, 4]. Preoperative standardized anti-tuberculosis drug treatment, timing of surgical intervention, and extent of debridement are also clearly specified in various guidelines [5, 6]. Complex spinal tuberculosis involving multiple vertebral segments is not uncommon in clinical practice. When spinal cord damage occurs rapidly and is severe, anti-tuberculosis treatment is often not performed before the operation to rescue spinal cord function in emergency surgery. Although “thorough debridement” is a relative concept, most scholars still require the complete removal of infected bone, sequestrum, necrotic intervertebral disc, abscess cavity wall, and inflammatory bone bridge in the diseased vertebral body [1, 3, 7, 8]. However, sometimes “complete debridement” cannot be achieved due to factors such as excessive lesion segments, insufficient tolerance of patients to long-term surgery, and high risk of vascular and nerve injury caused by thorough debridement of long segments. Conventional methods of anterior approach surgery to reconstruct stability include autologous bone grafts at the resection site, titanium cages combined with allogeneic bone grafts, and 3D-printed artificial vertebral bodies [9–12], which are all replaced by supports under the premise of resecting the diseased vertebral body. In this case, we adopted a method of “nonextreme” lesion debridement, free double fibular vertebrae-pelvic support, and exclusion of diseased vertebrae. We followed up with the patient for five years and achieved satisfactory results.

## Case presentation

A 60-year-old female patient was admitted to our spine surgery department with recurrent low back pain for six years and sudden onset of sensory-motor deficits in both lower limbs for 9 h. The patient had no previous history of pulmonary tuberculosis, cough, low-grade fever, night sweats, or other symptoms. The patient had no history of AIDS.

Physical examination: body temperature was 36.6 °C, anemic appearance, kyphotic deformity of the lumbar spine, and no reddening or sinus tract on the back skin, tenderness, and percussion pain at the vertex of lumbar kyphosis. Both lower limbs strength (Medical Research Council scale) was grade 0, the sensation of the perineum and both lower limbs was significantly decreased, absent reflexes in the lower limbs and anal, the sphincter was flaccid, and she was not possible to urinate voluntarily. The patient’s neurological status was diagnosed as grade B on the ASIA scale. The patient’s VAS score for back pain was as high as 7 points, and the ODI score for patient quality of life was 92 points. The patient’s nutritional score (Nutrition Risk Screening 2002, NRS 2002) was 3 points.

**Fig. 1:**
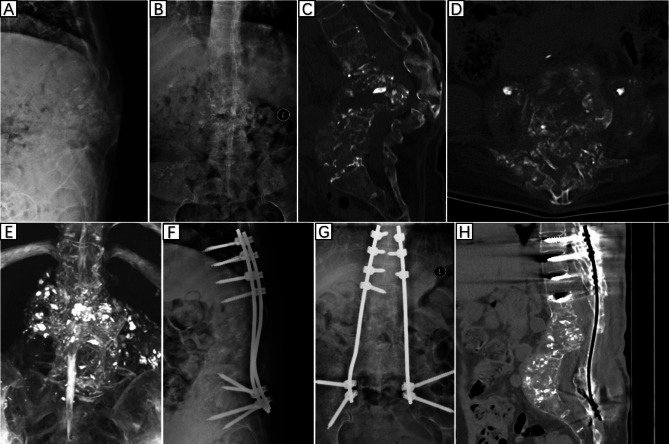
(A and B) Preoperative X-ray examination. (C-E) Preoperative CT examination. (F and G) Postoperative X-ray examination. (H) Postoperative CT examination

Laboratory and imaging examination: A blood test was performed after admission. White blood cells 2.26*10^9^/L, hemoglobin 87 g/L, platelet count 134*10^9^/L. Inflammatory markers were examined: CRP 13.23 mg/L and ESR 72 mm/h. Liver function examination showed albumin 32 g/L. HIV testing was negative. Chest X-ray and lung computed tomography (CT) revealed pleural effusion, and no signs of tuberculosis destruction were found in the lungs. Because the T12-L5 vertebral body was severely damaged, X-rays could not clearly show the structure of the lumbar spine (Fig. 1A, B). CT sagittal images showed severe destruction of all lumbar vertebral bodies, with the apex of lumbar kyphosis located at L2 and a kyphotic Cobb angle of + 68° (Fig. 1C). Free sequestrum was seen in the spinal canal, and the bony structures of the posterior columns of L1-L5 had been extensively eroded, with a large number of irregular cavities in the vertebral plate, part of the spinous processes, and the pedicles, which were interconnected with the cavities in the vertebral body, and the entire vertebral body was “honeycomb-shaped” (Fig. 1D). CT three-dimensional reconstruction imaging showed amorphous sediment-like sequestrum throughout and around the psoas major muscle (Fig. 1E). Magnetic resonance imaging (MRI) of the spine could not be completed because the patient could not tolerate prolonged lying down.

### Treatment process

**Fig. 2:**
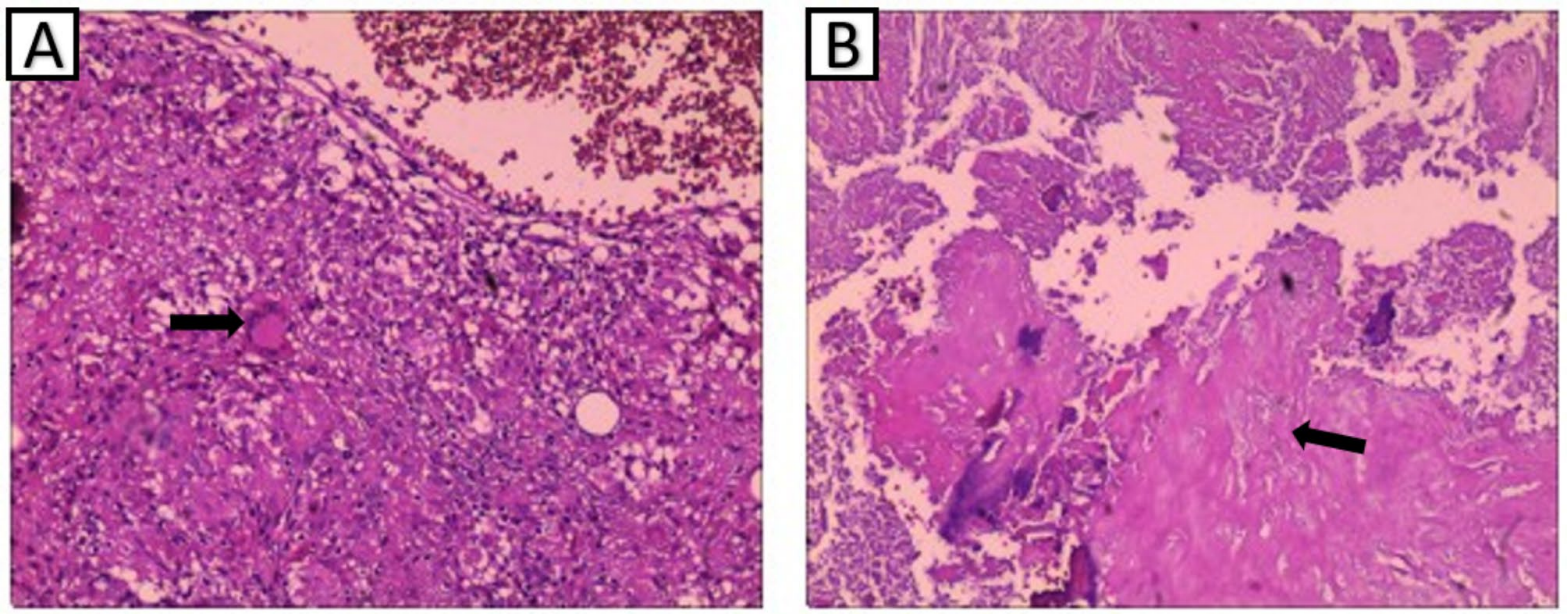
(A and B) Pathological examination after primary surgery. Figure A shows inflammatory granuloma, seen as multinucleated giant cells and epithelioid cells, as indicated by the arrows. Figure B shows extensive caseous necrosis, and the necrotic areas are visible microscopically as unstructured granular red-stained material

**Fig. 3:**
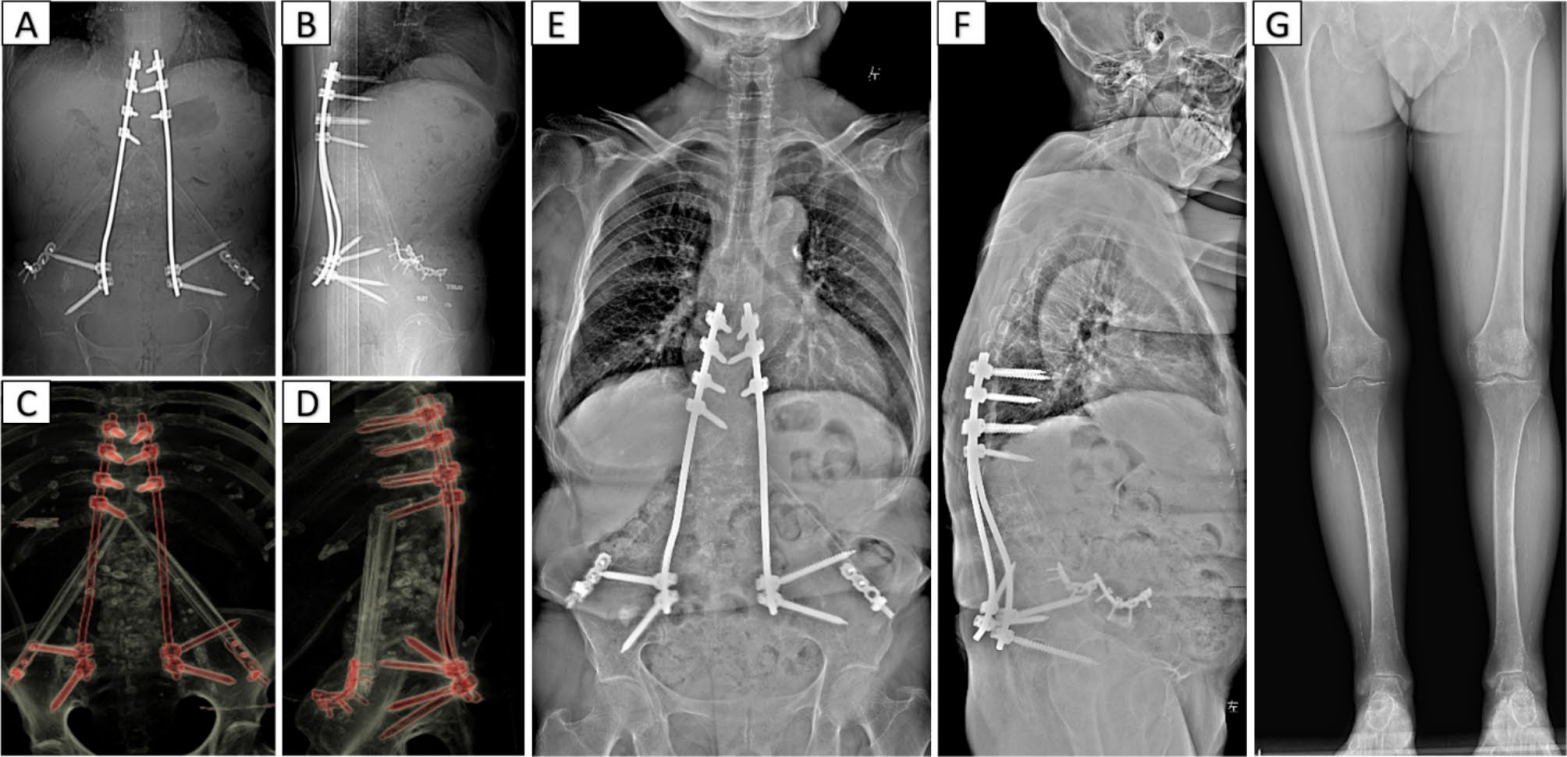
(A and B) Postoperative X-ray examination. (C and D) Postoperative CT examination. (E and F) The most recent postoperative X-ray examination of the full length of the spine. (G) X-ray examination of the full length of the lower limb after surgery showed partial absence of the fibula

**Fig. 4:**
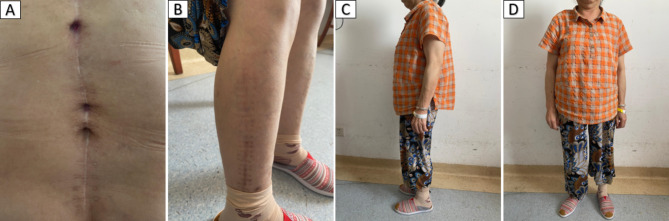
(A) The surgical incision on the patient’s back showed that 3 tuberculosis sinuses had healed. (B) Patient’s surgical scar at the removal of the fibula from the lower limb. (C and D) Image of the patient in the standing position during follow-up

**Fig. 5:**
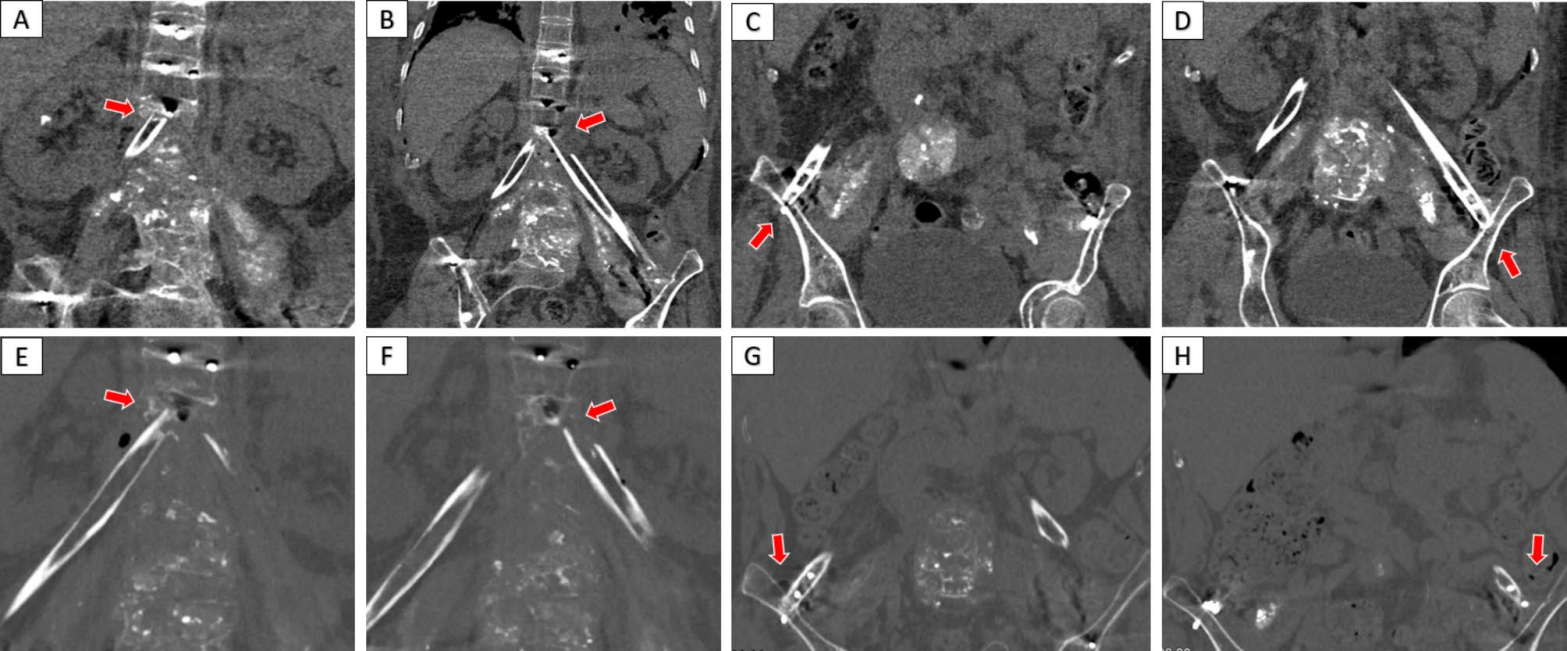
(A-D) After the second stage, CT reexamination showed that the bilateral fibula was fixed with the lower edge of the T12 vertebral body and the upper edge of the iliac spine. (E-H) During the 5-year postoperative follow-up period, there was no significant subsidence of the fibula at the fixation site compared to Figures A-D.

**Fig. 6:**
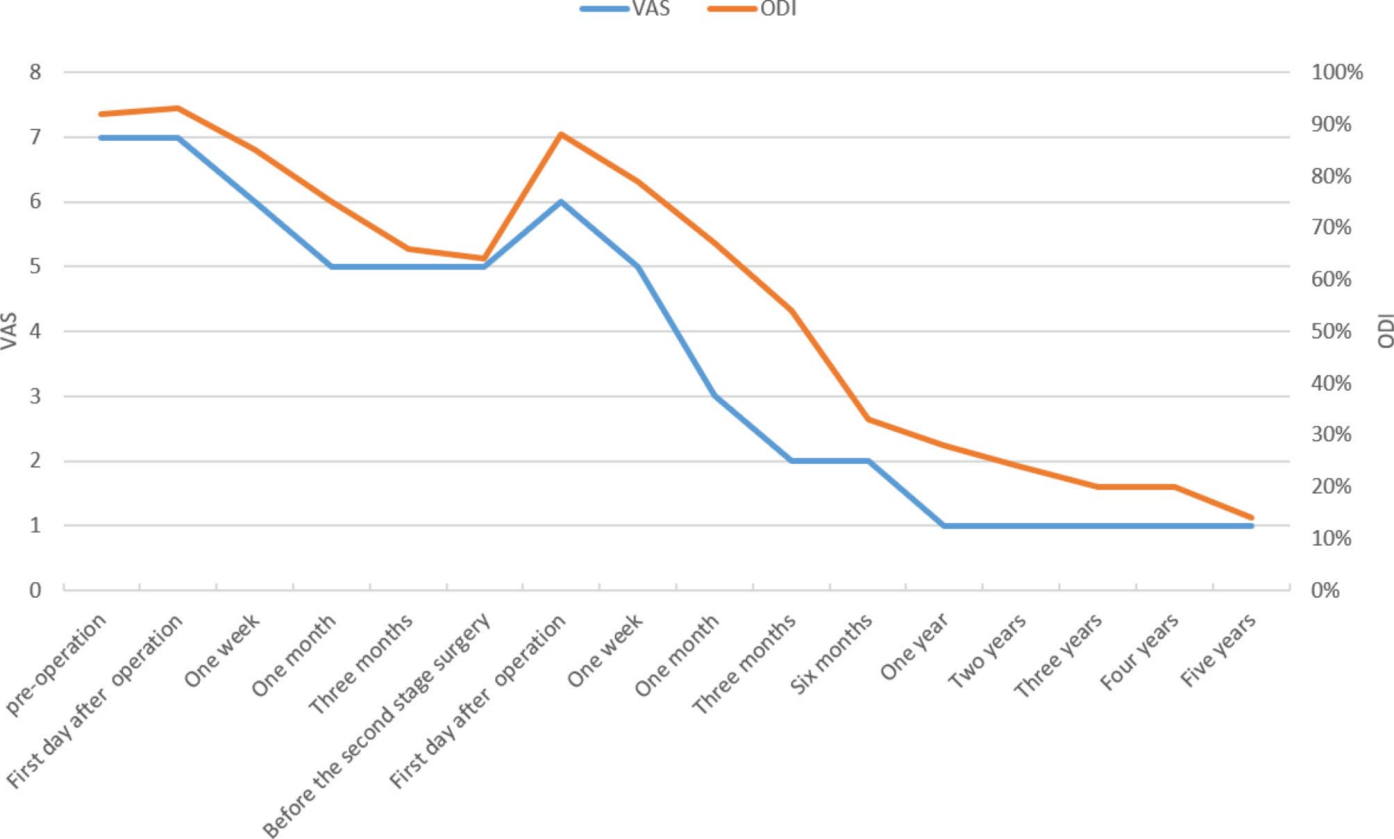
Patients’ VAS scores and ODI scores in the hospital and during the 5-year follow-up period

**Fig. 7:**
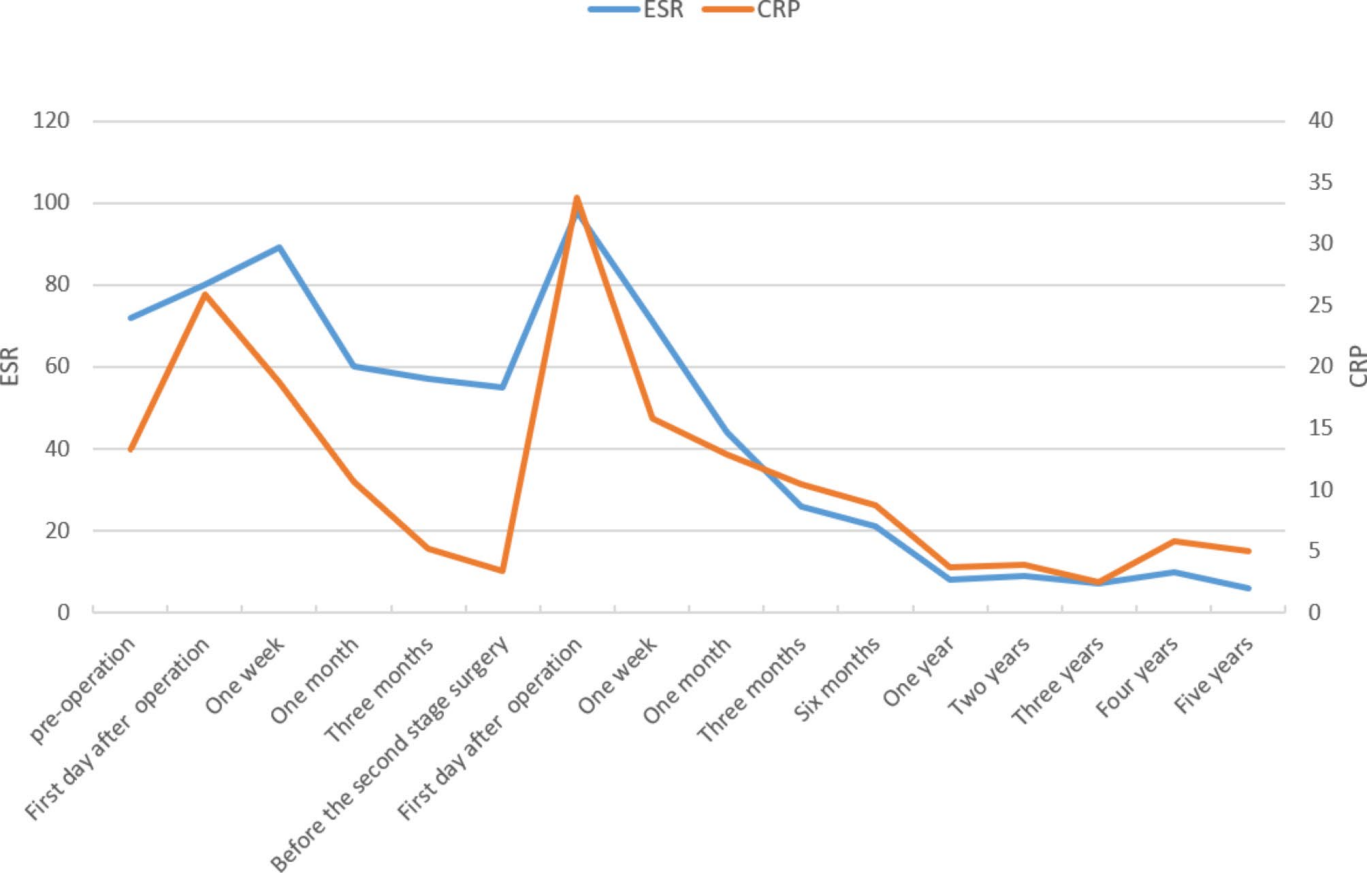
Patients’ ESR and CRP in the hospital and during the 5-year follow-up period

The patient was placed in the lateral position, and after the position of the abscess was located by ultrasound, the abscess was punctured through the right psoas major muscle to obtain 300ml of pus containing gravel-like sequestrum. The density of Mycobacterium tuberculosis in the pus smear was > 20,000/mm^2^, and pathological examination of the solid material after pus filtration revealed bone tuberculosis. Four anti-tuberculosis drugs (rifampicin, isoniazid, ethambutol, pyrazinamide) were given to the patient immediately after the diagnosis of bone tuberculosis was confirmed by puncture. Pleural effusion puncture fluid examination was normal. Due to acute paralysis, the day after the diagnosis of bone tuberculosis was confirmed by puncture, the patient underwent a posterior spinal approach to remove the paravertebral psoas abscess, laminectomy with spinal decompression, and long-segment spinal fixation surgery. The kyphotic Cobb angle was corrected to + 25°, and the spinal cord was decompressed (Fig. 1F-H). Intraoperative samples were taken and sent for pathological examination to reconfirm tuberculosis infection (Fig. 2A and B). The next day after surgery, the patient continued to take oral anti-tuberculosis drugs. The patient showed quadriceps femoris recovery on postoperative d3. The patient’s lower limb muscle strength and fecal and urinary functions improved significantly after 19 days. In the 4th postoperative week, three tuberculous sinus tracts appeared in the surgical incision in the lumbar back, draining approximately 12–15 ml of pus per day. The patient was re-admitted to our hospital after three months of regular and adequate anti-tuberculosis drug therapy and nutritional therapy at the local tuberculosis hospital. At this time, the patient’s lower limb sensation, muscle strength, reflexes, and fecal and urinary function were normal. The patient’s VAS score for back pain was 2 points for lying down and 5 points for standing up. The patient’s neurological status was diagnosed as grade E on the ASIA scale. The patient’s nutritional score (Nutrition Risk Screening 2002, NRS 2002) was 1 point. Three tuberculosis sinus tracts were observed at the surgical incision on the patient’s back, with secretion of 3 ml/24 hours. The patient’s ESR was 55 mm/h, CRP was 3.36 mg/L, hemoglobin concentration was 112 g/L, serum albumin was 39 g/L, and sinus tract secretion bacterial culture was negative. The patient underwent a two-stage transabdominal anterior approach to remove the abscess. Limited removal of lumbar lesions, and transperitoneal reconstruction of the pelvic-thoracic 12-vertebral body support using bilateral free fibulae were performed (Fig. 3A-D). Internal fixation remains stable as seen on recent follow-up (Fig. 3E-G). The amount of pus secreted by the sinus tract gradually decreased after the operation and healed completely after seven months (Fig. 4). Four anti-tuberculosis drugs (rifampicin, isoniazid, ethambutol, pyrazinamide) were given to the patient immediately after the diagnosis of bone tuberculosis was confirmed by puncture, with streptomycin added during the first 3 months of intensive period. Isoniazid and rifampicin during the consolidation period, for a total of 18 months of anti-tuberculosis treatment. During postoperative follow-up, the anti-tuberculosis treatment was effective. The patients were followed up for five years. After the second stage operation, the nutritional status of the patient continued to improve, and the tuberculosis sinus was healed after seven months. One year after the operation, the patient’s VAS score for back pain was 1 point, and the patient’s ESR and CRP levels returned to normal. Five years after the operation, the ODI of patient quality of life was 14 points. There was a 4-degree change in the Cobb angle over five years. During the five-year follow-up period, the grafted fibula did not experience any subsidence (Figs. 5, 6 and 7).

### Operation

To salvage spinal cord function, it is necessary to correct kyphosis in addition to decompressing the spinal canal to provide stronger fixation of the spine. At one stage, we opted for posterior spinal surgery and performed laminar decompression at the L1-L3 segments, removing pus and sequestrum within the spinal canal and anterior to the dura at the T12-L3 segments, as well as removing pus and part of the sequestrum within the paravertebral lumbar psoas major muscle. Pedicle screw fixation was performed on T9-11 bilaterally and the right side of T12, and the bone knife chiselled about 1 × 2 × 0.5 cm area of the posterior superior iliac spine, with distal and proximal points of entry, respectively, with the proximal screws oriented towards the anterior superior iliac spine, and the distal screws oriented towards the anterior inferior iliac spine. The goal of this procedure was not to pursue perfect lumbar correction, so after partial correction of the kyphotic deformity via transpedicular osteotomy of the posteriorly convex parietal vertebrae (L2), we fixed the lumbar spine in an acceptable kyphotic position. Except for the articular eminence, no additional autogenous iliac bone graft was performed. The distance from the middle of the T12 vertebral body to the upper edge of the pelvic iliac spine was measured to be 21.6 CM on CT imaging before the stage 2 surgery, and the median Stoppa approach was taken to expose the inner wall of the pelvis to the T12-L5 vertebral body extraperitoneal on both sides. The pus was aspirated out of the iliac fossa in bilateral iliac fossae of approximately 200 ml. After the first posterior debridement and three months of anti-tuberculosis treatment, the paravertebral pus thinned out and was replaced by a large amount of scar and sequestrum encased by scar. During the operation, the palpable free sequestrum was dissected and removed from the scar as much as possible, the lesion bone in the anterior part of the L1-L5 vertebral body was removed, the sclerotic bone in the cavity wall was abraded after exposing the cavity, accompanied by extensive oozing and instability of blood pressure, and most of the focal vertebral body of L1-L5 was absent. After measuring the graft distance again during the operation, 23 cm of bilateral nonvascularized fibula was taken, the lower end of the T12 vertebrae was perforated, one end of the fibula was inserted into the perforation from the lower edge of the T12 vertebrae, and the caudal end was inserted into the bone at the upper edge of the iliac spine for approximately 8 mm. The titanium plate was bent into a L shape, and the fibula was fixed to the bilateral iliac wings, and fixed with the assistance of a titanium plate for pelvic reconstruction. On the fifth postoperative day, the patient got out of bed and walked, and the patient reported that the symptoms of low back pain were significantly reduced when standing compared with those before surgery.

## Discussion

China has been listed as a country with a high global burden of tuberculosis in recent decades [13]. Spinal tuberculosis is the most common extrapulmonary tuberculosis, accounting for 1-3% of all tuberculosis and 50% of all bone and joint tuberculosis. It is the most common and serious form of tuberculosis damage in bones. Spinal tuberculosis usually presents as back pain; in severe cases, it also causes spinal cord nerve compression, kyphosis and spinal instability [14, 15]. For spinal tuberculosis combined with large paravertebral abscesses, kyphotic deformity, and spinal instability leading to nerve damage, surgery is required to remove the lesion, relieve nerve compression, correct the deformity, and reconstruct spinal stability [2, 16, 17]; however, the timing of surgery is highly controversial, and patients with severe preoperative anemia, high ESR, malnutrition, poor surgical tolerance, and inadequate preoperative anti-TB treatment are classified as surgical contraindications [3, 18, 19]. Correcting these adverse factors takes a certain amount of time, and for patients with combined acute nerve damage, the optimal time for surgery may be missed, which may even lead to severe consequences such as the inability of patients with nerve damage to recover. In 1935, Seddon [20] proposed through autopsy observation that spinal tuberculosis nerve damage was the result of compression and invasion of the spinal nerve by external forces such as dislocated and angulated vertebral bodies, pus, granulomas, and sequestrums. If the compression results in tethering of the anterior spinal arteries, permanent damage to the spinal cord hemorrhage could result. Thus, the risks associated with these preoperative contraindications are considered acceptable for patients with acute neurological damage [3, 21]. More studies are still needed to investigate the optimal timing of surgery in patients with complex spinal tuberculosis.

In this case, the patient’s paralysis occurred rapidly, CT showed incomplete dislocation of L2 kyphosis, multiple vertebral pedicles were invaded by tuberculosis, the spine was severely unstable, and the L2-L3 spinal cord was compressed by sequestrum from the front. Although the patient failed to complete the MRI, we suspected that the spinal cord compression should be higher than L2 according to the plane of paralysis, and during surgery, we found that the abscess had spread to the T12 plane, which confirmed our conjecture. After adequate evaluation of the patient’s general condition and surgical tolerance by consultation in multiple disciplines, including anesthesiology, nutrition, and infectious diseases, we performed emergency surgery and minimized the occurrence of related complications by blood transfusion and injection of albumin, streptomycin, and isoniazid before and during surgery. Fortunately, no adverse complications occurred. The patient showed quadriceps femoris recovery on postoperative d3. Through anti-tuberculosis treatment, nutritional support, and rehabilitation training, the patient’s lower limb muscle strength and fecal and urinary function completely recovered after 19 days. The patient started walking with the help of crutches. However, the patient complained of severe low back pain when standing and walking, which had to be relieved by lying in bed after transient walking activities. The second stage of surgery was delayed by two months because of anemia and hypoproteinemia. The operation’s aim was thorough anterior debridement and reconstruction of the anterior column of the spine. Debridement methods that completely remove abscesses, tuberculosis granulation tissue, necrotic intervertebral discs, sequestrums, and sclerotic bone that abrade the cavity wall in tuberculosis lesions have now been accepted by the vast majority of surgeons [1, 3, 7]. Incomplete debridement means a delayed treatment time, sinus tract formation, a high recurrence rate, and a high failure rate of anterior fusion [3, 22]. However, in the case of this patient, achieving the extent and standard of complete debridement meant removing all of the patient’s lumbar vertebrae, which was very difficult to achieve. Similarly, anterior reconstruction at such a long level is very difficult. The most commonly used bone graft materials for STB surgery are allogenic bone grafts, autologous iliac bone grafts, and titanium mesh cages filled with allogenic or autologous bone [23]. However, for such a long-segment anterior reconstruction, titanium cage mesh filled with conventional iliac bone grafting cannot maintain its stability. The fibula, rib, and iliac bones are commonly considered donors for bone grafting. Rib grafts are commonly used for anterior and posterior spinal fusion at the thoracolumbar level due to their inherent angulation [24]. However, rib grafts have a relatively thin cortex and relatively poor stability, and therefore often require additional fixation. Mesh filled with bone graft is prone to dislocation and downward settling at a later stage [25]. Implantation of such a long titanium cage mesh is prone to failure of fusion and is more costly. Iliac bone grafts also have limitations, including inadequate length and potential persistent donor-site pain [24]. While the fibula has significant advantages in its length, strength of support, and limited donor site morbidity, it can be adapted to different positions and lengths of the vertebral column, and the fibula can be harvested up to 25 cm [26]. Therefore, in this patient with long-segment anterior reconstruction, we preferred fibula extraction. Winters et al. [27–29] described the fibula as osteotomized and folded in a double-, triple-, or quadruple-barrel construction to increase the strength of the reconstruction. In this patient, we used bilateral fibula implants to increase the strength and stability of the reconstruction. During the five-year follow-up period, the grafted fibula did not experience any subsidence. After the two-stage operation, the nutritional status of the patients continued to improve, and the quality of life also increased greatly. The patient was satisfied with the treatment.

To our knowledge, this is the first instance of complicated spinal tuberculosis being treated with a long-segment fibula transplant, and the patient was satisfied with the surgical results after five years of follow-up.

## Conclusions

For patients with spinal tuberculosis and acute paralysis, it is essential to relieve spinal cord compression as soon as possible to recover spinal cord function. For lesions that cannot be debrided entirely, although limited debridement combined with anti-tuberculosis drug therapy has the risk of sinus formation and tuberculosis recurrence, it is much safer than the risk of thorough debridement surgery. In this case, an unconventional long-segment fibula graft, pelvis-vertebral support, was an effective reconstruction method.

## Data Availability

The authors declare that data supporting the findings of this study are available within the article.

## Abbreviations

STB: Spinal tuberculosis
ASIA: American Spinal Injury Association
VAS: Visual analog scale
ESR: Erythrocyte sedimentation rate
CRP: C-reactive protein
ODI: Oswestry disability index
MRI: Magnetic resonance imaging
CT: Computed tomography
